# Ezrin Is a Novel Protein Partner of Aquaporin-5 in Human Salivary Glands and Shows Altered Expression and Cellular Localization in Sjögren’s Syndrome

**DOI:** 10.3390/ijms22179213

**Published:** 2021-08-26

**Authors:** Clara Chivasso, Carl Johan Hagströmer, Kristie L. Rose, Florent Lhotellerie, Lionel Leblanc, Zhen Wang, Stefania Moscato, Clément Chevalier, Egor Zindy, Maud Martin, Benoit Vanhollebeke, Françoise Gregoire, Nargis Bolaky, Jason Perret, Chiara Baldini, Muhammad Shahnawaz Soyfoo, Letizia Mattii, Kevin L. Schey, Susanna Törnroth-Horsefield, Christine Delporte

**Affiliations:** 1 Laboratory of Pathophysiological and Nutritional Biochemistry, Université Libre de Bruxelles, 1070 Brussels, Belgium; Clara.Chivasso@ulb.be (C.C.); Florent.Lhotellerie@ulb.be (F.L.); Lionel.Leblanc@ulb.be (L.L.); Francoise.Gregoire@ulb.be (F.G.); Nargis.Bolaky@ulb.ac.be (N.B.); Jason.Perret@ulb.be (J.P.); 2 Division of Biochemistry and Structural Biology, Lund University, 221 00 Lund, Sweden; carl_johan.hagstromer@biochemistry.lu.se; 3 Department of Biochemistry, Vanderbilt University School of Medicine, Nashville, TN 37240, USA; kristie.rose@Vanderbilt.Edu (K.L.R.); zhen.wang@Vanderbilt.Edu (Z.W.); k.schey@Vanderbilt.Edu (K.L.S.); 4 Department of Clinical and Experimental Medicine, University of Pisa, 56126 Pisa, Italy; stefania.moscato@unipi.it (S.M.); chiara.baldini74@gmail.com (C.B.); letizia.mattii@med.unipi.it (L.M.); 5 Center for Microscopy and Molecular Imaging (CMMI), 6041 Gosselies, Belgium; clement.chevalier@hotmail.fr (C.C.); Egor.Zindy@ulb.be (E.Z.); 6 Laboratory of Neurovascular Signaling, Université Libre de Bruxelles, 6041 Gosselies, Belgium; Maud.Martin@ulb.be (M.M.); Benoit.Vanhollebeke@ulb.be (B.V.); 7 Department of Rheumatology, Erasme Hospital, Université Libre de Bruxelles, 1070 Brussels, Belgium; Muhammad.Shah.Soyfoo@ulb.be

**Keywords:** Sjögren’s syndrome, aquaporin-5, ezrin, salivary glands, protein–protein interaction

## Abstract

Sjögren’s syndrome (SS) is an exocrinopathy characterized by the hypofunction of salivary glands (SGs). Aquaporin-5 (AQP5); a water channel involved in saliva formation; is aberrantly distributed in SS SG acini and contributes to glandular dysfunction. We aimed to investigate the role of ezrin in AQP5 mislocalization in SS SGs. The AQP5–ezrin interaction was assessed by immunoprecipitation and proteome analysis and by proximity ligation assay in immortalized human SG cells. We demonstrated, for the first time, an interaction between ezrin and AQP5. A model of the complex was derived by computer modeling and in silico docking; suggesting that AQP5 interacts with the ezrin FERM-domain via its C-terminus. The interaction was also investigated in human minor salivary gland (hMSG) acini from SS patients (SICCA-SS); showing that AQP5–ezrin complexes were absent or mislocalized to the basolateral side of SG acini rather than the apical region compared to controls (SICCA-NS). Furthermore, in SICCA-SS hMSG acinar cells, ezrin immunoreactivity was decreased at the acinar apical region and higher at basal or lateral regions, accounting for altered AQP5–ezrin co-localization. Our data reveal that AQP5–ezrin interactions in human SGs could be involved in the regulation of AQP5 trafficking and may contribute to AQP5-altered localization in SS patients

## 1. Introduction

Sjögren Syndrome (SS) is a chronic autoimmune disease characterized by the lymphocytic infiltration and destruction of exocrine glands, including salivary and lacrimal glands. Sicca symptoms are the main clinical manifestation of SS. Apoptosis of salivary gland epithelial cells (SGECs) and the abnormal expression and localization of aquaporin-5 (AQP5) have been proposed to play roles in the impairment of the secretory function of salivary glands (SGs) in SS.

AQP5 is a water channel belonging to the family of aquaporins [1,2], which is expressed in the acinar cells of SGs and plays a key role in saliva secretion [3,4]. The current model of saliva secretion relies on a two-step mechanism: the first step allows the secretion of a primary isotonic fluid by the acinar cells, and the second step involves modification of the primary fluid composition by the ductal cells, finally leading to hypotonic saliva entering the mouth [5,6]. Proper AQP5 translocation to the apical plasma membrane of acinar cells is therefore essential for saliva production. Protein–protein interactions have been shown to be involved in the regulation of the AQP’s transcellular water permeability, involving both gating and intracellular trafficking [7]. It has been shown that AQP5 trafficking occurring in response to acetylcholine in SGs [8,9] involves its C-terminal region [10,11], and both its gating and trafficking may depend on transient protein–protein interaction [7]. Several proteins have been identified to date to interact with AQP5 and is associated with its trafficking and/or function such as PIP [11,12], TRPV4 [13], CA12, and NKCC1 [14]. Furthermore, AQP5 localization is altered in patients with SS [15,16] and mice models of SS [17,18,19].

Ezrin, a membrane-bound cytoskeleton linker protein of about 80 kDa, is a protein belonging to the family of ezrin/radixin/moesin (ERM) proteins. The ezrin/radixin/moesin (ERM) proteins are a family of actin-binding proteins that play central roles in endocytosis, phagocytosis, vesicular trafficking, and vesicle maturation by reorganizing the actin cytoskeleton [20]. ERM proteins regulate the membrane protein trafficking of several proteins such as NHE3 [21], the proton pump H/K ATPase [22], NKCC2 [23], and the well-described aquaporin-2 [24]. ERM proteins act as a scaffold to facilitate signal transduction, leading to cell survival, proliferation, adhesion, and migration [25,26]. ERM proteins contain three functional domains: a highly conserved N-terminal FERM (four-point-one ezrin, radixin, moesin) domain, a central alpha-helical domain predicted to form coiled coils, and a C-terminal domain capable of binding actin [27]. The FERM domain is known to interact with amphipathic helical segments from several proteins [28], thereby forming a link between these proteins and the actin cytoskeleton [29]. Several crystal structures of such FERM–peptide complexes exist, including the crystal structures of the radixin and moesin FERM domains in complex with a peptide from the Na^+^/H^+^ exchanger regulatory factor (NHERF) [30,31] and the Merlin FERM domain in complex with its C-terminal domain, and reveal the structural basis for these interactions. As for what is known for aquaporins, AQP2 interacts directly through its C-terminus and the ezrin FERM. It should be noted that ezrin knockdown was linked to increased AQP2 membrane accumulation and reduced AQP2 endocytosis [24]. Moreover, ezrin knockout mice develop several alterations in the apical regions of intestinal and retinal epithelial cells, suggesting its essential role for the function and morphogenesis of epithelial cells [32], leading to post-weaning lethality, making functional studies on adult mice impossible [33].

The aims of this study were to investigate the existence of protein–protein interactions between AQP5 and ezrin in human SGs and to assess whether the abnormal localization of AQP5 could result from altered ezrin expression and localization. Furthermore, as the ezrin FERM domain is known to interact with other proteins, including the C-terminal region of AQP2, we hypothesized that the ezrin FERM domain binds the AQP5 C-terminus and explored this using computer modeling and in silico docking.

## 2. Results

### 2.1. Evidencing AQP5–Ezrin Complexes

**Figure 1:**
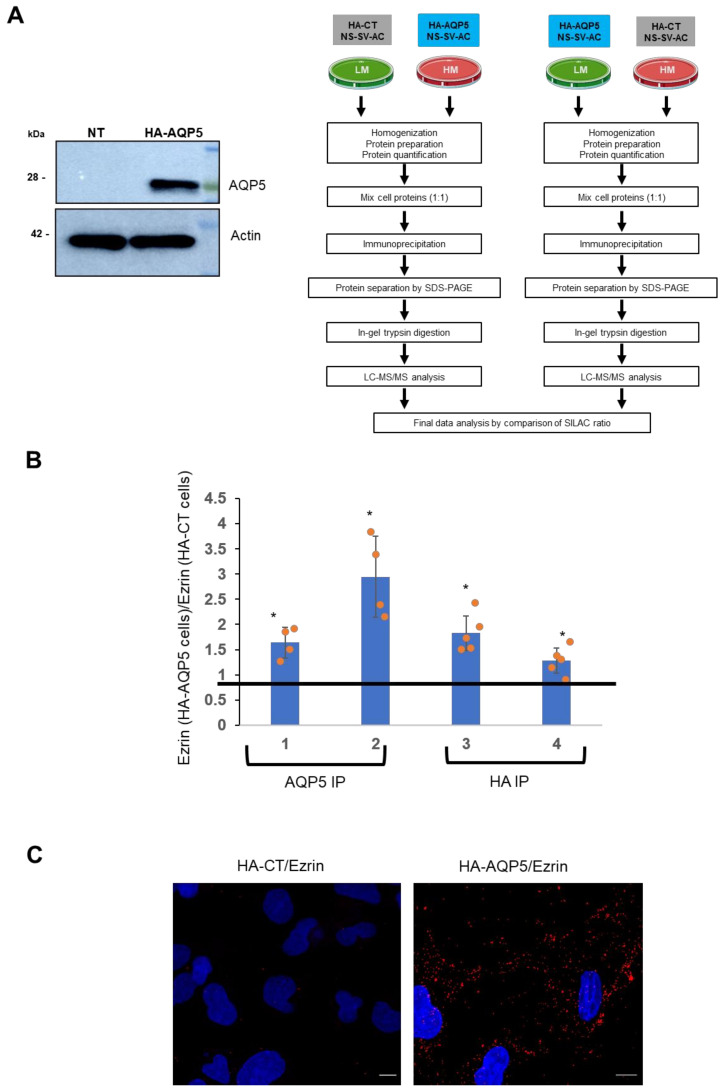
(**A**) Left, validation of NS-SV-AC transfection with HA-AQP5 plasmid. Right, schematic SILAC methodological workflow. NS-SV-AC cells grown in light (LM) or heavy (HM) medium were transfected with either HA-CT or HA-AQP5 plasmid constructs. Total proteins were prepared from the cells and mixed at a 1:1 ratio prior to immunoprecipitation and LC-MS/MS. (**B**) Immunoprecipitated proteins obtained using anti-AQP5 (samples 1 and 2) or anti-HA antibodies (samples 3 and 4) were subjected to SDS-PAGE, in-gel trypsin digestion, and LC-MS/MS analysis. The ratios of ezrin peptide intensities from AQP5-expressing cells (HA-AQP5) to control cells (HA-CT) were averaged (individual ratios shown as orange points). A one-sample t-test was used to determine if the mean ratio was different from 1 (expected for no interaction), * = *p* < 0.05. Samples 1 and 3: cells containing heavy isotope were transfected with tagged AQP5 (HA-AQP5) and cells containing light amino acid were transfected with the control construct (HA-CT). Labels were swapped for samples 2 and 4. (**C**) PLA of NS-SV-AC transfected with HA-CT (negative control as NS-SV-AC cells are devoid of endogenous AQP5 expression) or HA-AQP5. Interactions are represented by red spots (Texas Red) and nuclei are stained in blue. Scale bars correspond to 10 µm.

Protein–protein interactions between AQP5 and ezrin were established in the NS-SV-AC human cell line transfected with HA-CT or HA-AQP5 plasmids after 10 passages in a Stable Isotope Labeling with Amino Acids in Cell Culture (SILAC) medium. The efficiency of AQP5 transfection was verified by Western blot analysis (Figure 1A). When the light and heavy labels were swapped, a total of 131 and 59 proteins exhibited an inverse SILAC ratio following immunoprecipitation with anti-HA and anti-AQP5 antibodies, respectively. Among the 40 proteins immunoprecipitated by both anti-HA and anti-AQP5 antibodies, LC-MS/MS data analysis revealed the immunoprecipitation of AQP5 and ezrin, thus revealing for the first time a new AQP5 binding partner. Multiple ezrin peptides were detected and quantified, showing, in nearly all cases, higher intensities for the HA-AQP5-immunoprecipitated samples with both anti-AQP5 (samples 1 and 2) and anti-HA (samples 3 and 4) antibodies (Figure 1B). The intensities of light and heavy Arg/Lys-containing peptides inverted when SILAC labels were swapped between HA-CT- and HA-AQP5-transfected cells, indicative of a specific interaction between AQP5 and ezrin. Note that all data in Figure 1B were plotted as the ratios of intensities of ezrin peptides from AQP5-expressing cells to control cells, and these ratios are a significantly different from one (expected for noninteracting proteins).

**Figure A1:**
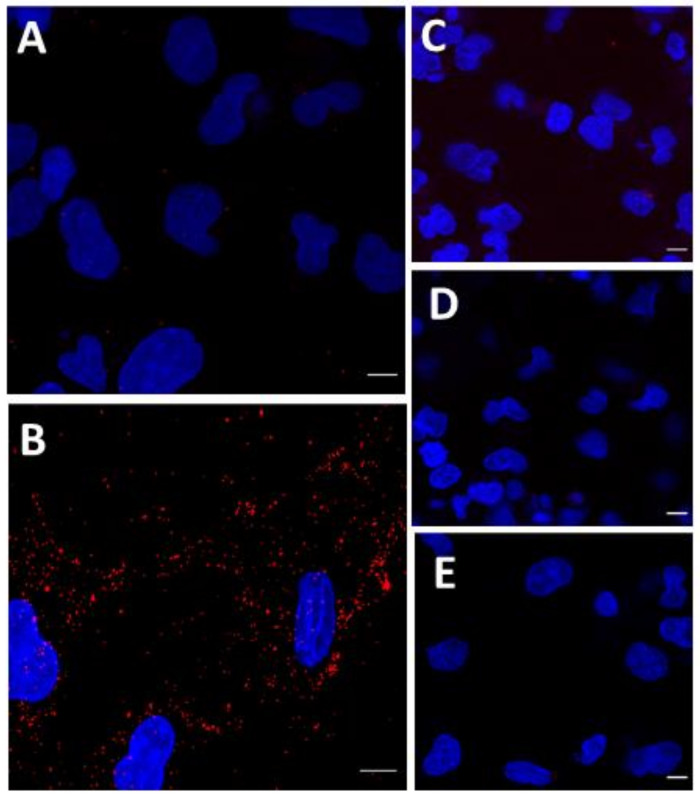
Protein–protein interactions visualized as red fluorescent spots on transfected NS-SV-AC with HA-CT (**A**) or HA-AQP5 (**B**). Negative controls were performed using NS-SV-AC transfected with HA-AQP5 in the absence of anti-HA antibody (**C**), anti-Ezrin (**D**), or both primary antibodies (**E**). Scale bar, 10 µm.

The existence of the AQP5–ezrin complexes was further confirmed by PLA in NS-SV-AC cells transfected with HA-AQP5 plasmid. The presence of red dots detected in the cytoplasm and membrane of HA-AQP5 NS-SV-AC cells, but not in HA-CT-transfected NS-SV-AC cells (used as a negative control as NS-SV-AC cells are devoid of endogenous AQP5 expression), results from distances between AQP5 and ezrin protein less than 40 nm and indicative of AQP5–ezrin complexes (Figure 1C). Appropriate negative controls are shown in Appendix A Figure A1.

### 2.2. Computer Modeling of AQP5–Ezrin Interaction

**Figure 2:**
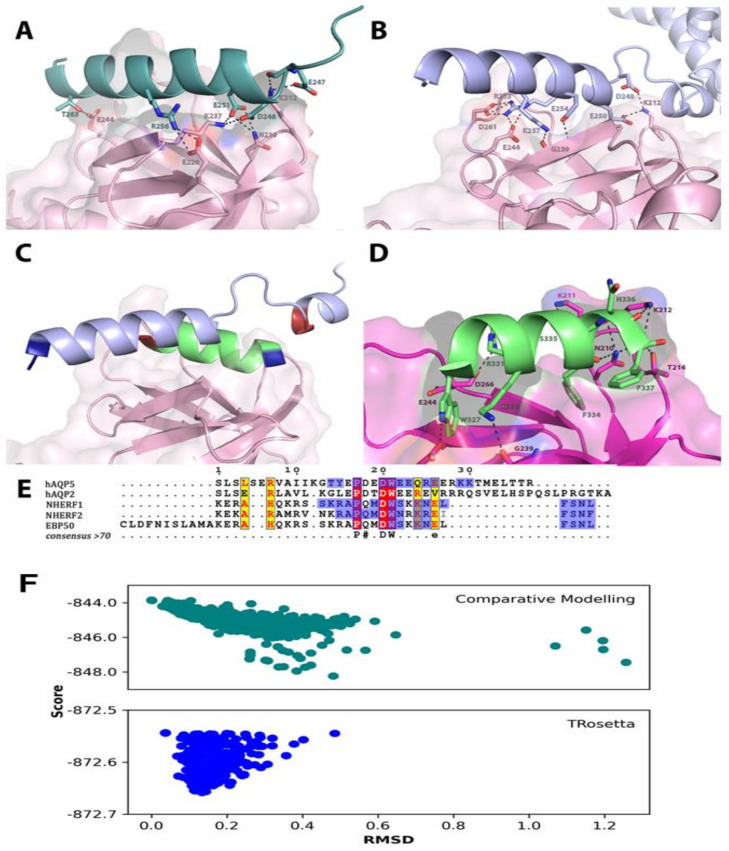
(**A**) Docking solution for complex between AQP5 C-terminus (model 1) and human ezrin FERM domain (PDB code 1NI2) in pink. Residues predicted to form polar contacts by PyMOL are shown with hydrogen bonds as dotted lines. (**B**) AQP5 C-terminus (model 2) by TrRosetta shown in blue. (**C**) Overlay of the AQP5 C-terminus model 2 (blue) with complex between the NHERF-2 C-terminal peptide (green) and the radixin FERM domain (magenta) (PDB code 2D11). The N-and C-termini of the peptide binding regions are shown in blue and red, respectively. The peptides bind in a similar way but in the opposite direction. (**D**) Zoom-in on the interaction site in the radixin FERM–NHERF-2 peptide complex showing the involvement of the same residues found in predicted complex between AQP5 model 2 and human ezrin FERM domain. (**E**) Multiple sequence alignment between C-terminus of human AQP5, AQP2, Drosophila NHERF-1 and NHERF-2, and human SBP50. Conserved residues are highlighted in yellow (highly conserved) and red (fully conserved). Residues involved in the interaction with FERM domains are in blue. (**F**) Scoring of initial AQP5–ezrin interactions generated with PyRosetta. Lower score and RMSD are preferred. The best scoring solutions were studied further using HADDOCK 2.4.

**Figure 3:**
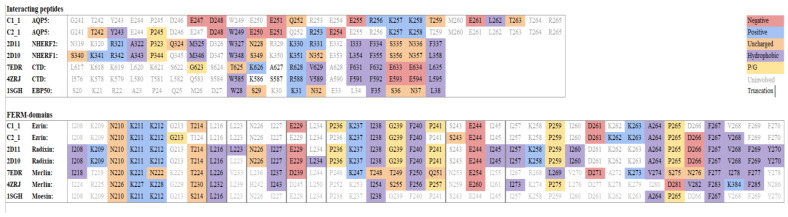
Comparison of interacting residues in FERM–peptide complexes. Residues involved in the interaction in the interacting peptides, as well as the FERM domains, were identified using PRODIGY and highlighted in color according to the type of residue. Grey lines indicate truncations of the sequences for visual purposes. The used sequences are as follows: C1_1, AQP5 C-terminus model 1 (comparative modeling) with human ezrin FERM; C2_1, AQP5 C-terminus model 2 (TrRosetta) with human ezrin FERM; 2D11 and 2D10, Mus musculus radixin FERM with NHERF-2 and NHERF-1 peptides, respectively; 7EDR, Drosophila melanogaster Merlin FERM with Merlin C-terminal domain; 4ZRJ, Human Merlin with C-terminal domain; 1SGH, Human Moesin FERM with EBP-50 (also known as NHERF-1).

The putative interaction between the AQP5 C-terminus and the ezrin FERM-domain was explored using computer modeling and in silico docking. First, as the AQP5 crystal structure is disordered after Pro 246, Robetta was used to generate structural models of the complete AQP5 C-terminus [34]. Two different approaches were used: (1) comparative modeling based on the human AQP5 crystal structure [35] (PDB code 3D9S) and (2) de novo structural prediction using TrRosetta. In both models, the C-terminus was predicted to form an α-helix; however, the second approach generates a C-terminus that is seemingly more flexible (Figure 2A,B). Initial docking runs were performed using PyRosetta [36], which generated 1000 decoys. These decoys were scored, and the best scoring decoys were inspected for potentially interacting residues. These residues were then used as input for HADDOCK 2.4, [37] which generated 108 structures for model 1 (comparative modeling) grouped into 9 clusters, and 147 structures for model 2 (TrRosetta) grouped into 9 clusters (Figure 2F and Table 1). The top scoring clusters of both docking runs (Cluster1_1 for model 1 and Cluster2_1 for model 2) were further analyzed in PyMOL (Figure 2A,B) and PRODIGY [38] (Table 2 and Figure 3). Both docking solutions showed the helical AQP5 C-terminus interacting with the same part of the ezrin FERM-domain; a groove formed between two β-sheets within the sub-domain C β-sandwich (Figure 2A,B) that has been shown to be a binding site for other helical peptides, exemplified by the radixin FERM–NHERF peptide complex [30] (Figure 2C,D). Interestingly, in both docking models, the AQP5 C-terminus binds in the opposite direction, but nevertheless involves the same ezrin residues as seen in other FERM-peptide crystal structures (Figure 3). In contrast, the AQP5 residues proposed to take part in the interaction differs between the two different C-terminal models (Figure 3). Specifically, in model 1, interacting residues along the entire predicted C-terminal helix are proposed by both PyMOLl (Figure 2A) and PRODIGY (Figure 3). In contrast, the more flexible C-terminus in model 2 interacts mainly with the proximal part of the predicted C-terminal helix (Figure 2B). When comparing the two docking solutions with the crystal structure of the radixin FERM–NHERF peptide complex, cluster2_1 (model 2) emerges as the most similar model (Figure 2C,D). In particular, the residues proposed to participate in the interaction are highly conserved between the AQP5 C-terminus in cluster2_1 and the NHERF peptide, as well as other peptides known to interact with the same site on FERM-domains (Figure 2E and Figure 3). Based on this, we propose that cluster2_1 represents the most likely model of the ezrin FERM–AQP5 complex (Figure 2B).

### 2.3. In Vivo Altered Expression and Localization of AQP5–Ezrin Complexes, AQP5, and Ezrin in SGs from SS Patients

**Figure 4:**
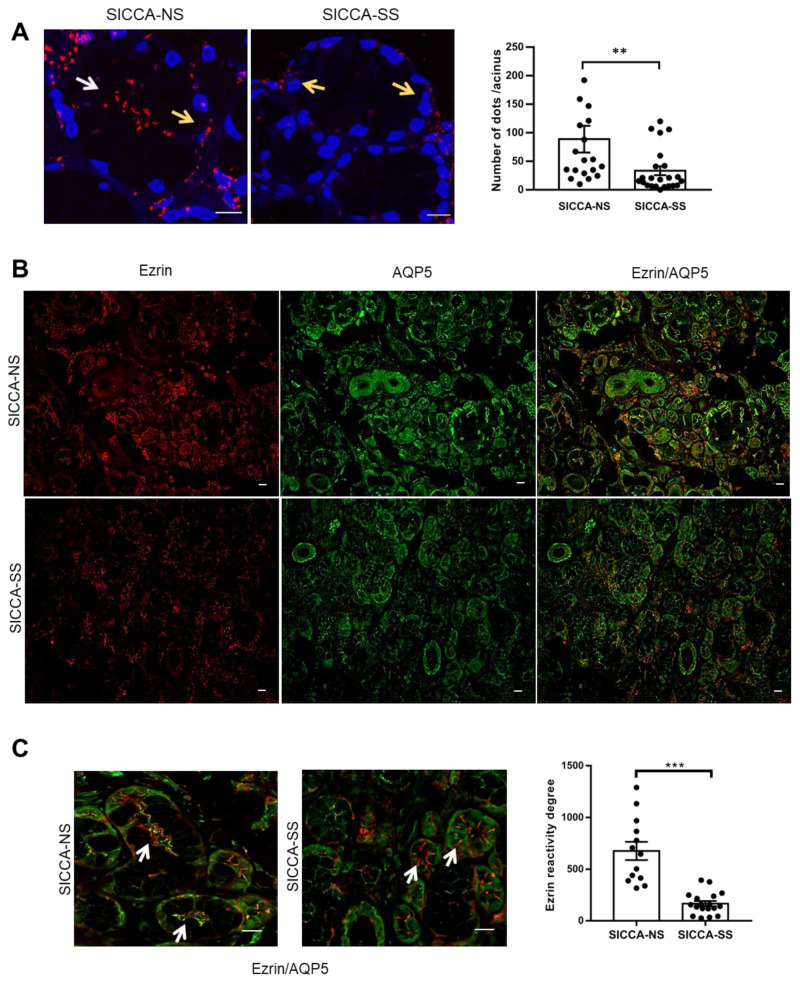
(**A**) Left, PLA on hMSG biopsies from SICCA-NS and SICCA-SS. Nuclei were labeled with DAPI (blue) and interactions are represented by red spots. White and yellow arrows indicate the apical and basal region, respectively. Scale bar, 10 µm. Right, quantification of PLA red spots per acinus. Results are expressed as the mean ± S.E.M cells (*n* = 19 SICCA-NS, 23 SICCA-SS). Statistical analysis was performed using the Mann–Whitney U test; ** *p* = 0.0017. (**B**) Representative immunofluorescence images of AQP5 (green) and ezrin (red) in SICCA-NS and SICCA-SS hMSG. Scale bar, 20 µm. (**C**) Left, blow-up of the B panel squares showing merged channels (red—ezrin, green—AQP5, and yellow—ezrin/AQP5). Scale bar, 20 µm. White arrows indicate the ezrin localization in the apical (SICCA-NS) and lateral (SICCA-SS) regions. Right, semiquantitative evaluation of ezrin expression. Results are expressed as the mean ± S.E.M. (*n* = 13 SICCA-NS, *n* = 18 SICCA-SS). Statistical analysis was performed using the Mann–Whitney U test, *** *p* < 0.001.

**Figure A2:**
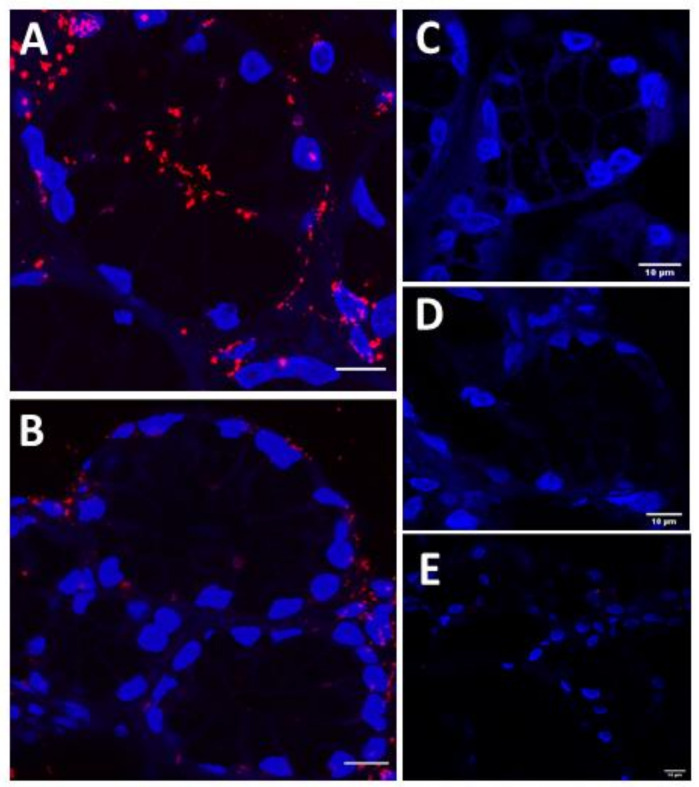
Protein–protein interactions visualized as red fluorescent spots in hMSG from SICCA-NS (**A**) and SICCA-SS (**B**). Negative controls were performed using hMSG from SICCA-NS in the absence of anti-AQP5 antibody (**C**), anti-Ezrin (**D**), or both primary antibodies (**E**). Scale bar, 10 µm.

AQP5–ezrin complexes were assessed by PLA in hMSG biopsies from patients presenting sicca symptoms but without evidence of autoimmunity suggestive of Sjögren’s syndrome (SICCA-NS; used as control) and patients presenting sicca symptoms and Sjögren’s syndrome (SICCA-SS). The number of AQP5–ezrin complexes was significantly reduced in SICCA-SS as compared to SICCA-NS (*p* = 0.0017). Furthermore, while the AQP5–ezrin complexes were mainly localized at the apical region of SICCA-NS hMSG acinar cells, they were mostly absent in SICCA-SS hMSG acinar cells (Figure 4A). Appropriate negative controls are shown in Appendix A Figure A2.

Double immunofluorescence on hMSG biopsies were performed to identify the regions of ezrin and AQP5 co-localization (Figure 4B). Our data showed that SICCA-NS hMSG acinar cells displayed strong positive co-localized ezrin and AQP5 staining at the apical membrane of acinar cells (yellow in merged pictures). In contrast, in SICCA-SS hMSG acinar cells, ezrin immunoreactivity was decreased at the acinar apical region and higher at basal or lateral regions, accounting for altered AQP5–ezrin co-localization. Semiquantitative analysis showed a significant reduction in ezrin expression in SICCA-SS compared to SICCA-NS tissues (Figure 4C).

## 3. Discussion

AQP5 is a major player in saliva secretion due to its involvement in water transport across the acinar apical plasma membrane. As with AQP2, AQP5 is localized in cytoplasmic vesicles that can translocate to the apical plasma membrane in response to hormonal stimuli. AQP5 trafficking is regulated by various mechanisms that involve not only a post-translation modification but increasingly its interactions with protein partners, as observed for other AQPs. Furthermore, altered AQP5 localization has been documented in hMSG from SS patients [15,16] and SG from SS mice models [17,18,19]. On the other hand, ezrin acts as a linker between the cytoskeleton and the plasma membrane and plays a role in the maintenance of the SG acinar cell architecture [39], cell polarity, and cell migration, contributing to the immune response and tumor progression [40,41]. In this study, we showed for the first time the existence of protein–protein interactions between AQP5 and ezrin, in vitro in a human salivary gland cell line (NS-SV-AC cells), and in vivo in human SGs. We further assessed whether the abnormal localization of AQP5 could result from altered ezrin expression and localization. Pulldown and co-immunoprecipitation experiments have shown ezrin as a protein partner of AQP2 in Madin Darby Canine Kidney (MDCK) cells. The protein–protein interaction was mediated by direct contact between the C-terminal region of AQP2 and the N-terminal FERM domain of ezrin and facilitated AQP2 endocytosis [24]. Another study showed the interaction between the C-terminal domain of AQP0 and ezrin FERM domain in lens fiber cells [42]. It should be noted that AQP0 is the most abundant membrane protein in the lens and plays important roles in the maintenance of lens transparency and homeostasis functioning as a water channel. In our study, PLA and SILAC coupled to immunoprecipitation and mass spectrometry analysis showed for the first time that AQP5 is also capable of interacting as well with ezrin in human NS-SV-AC cells. Using computer modeling and in silico docking, we propose a model whereby AQP5 interacts with the ezrin FERM domain via a helix formed by the AQP5 C-terminus. The comparison with crystal structures of other complexes between FERM domains and helical peptides reveals significant similarities, particularly concerning the binding surface on the FERM domain (Figure 3). Moreover, a multiple-sequence alignment between the NHERF-peptides known to interact with radixin and moesin FERM domains and the C-termini from AQP2 and AQP5 reveal a high degree of sequence conservation.

Interestingly, several residues involved in complex formation with the FERM domains are found amongst the conserved residues, suggesting a shared mode of interaction. Based on this comparison, we propose a novel consensus motif for the interaction between FERM domains and helical peptides: PXXDWXX(X)R/KXE. This motif is a modification of the previously proposed motif for the interaction between NHERF-peptides and the radixin/moesin FERM domains (MDWXXXXX(L/I)FXX(L/F)) and involves the same peptide region [30]. Interestingly, despite these similarities, our docking model of AQP5-FERM shows the helical peptide binding in the opposite direction compared to previous FERM-peptide structures. Further studies will be needed to elucidate whether this is an artefact of the docking or a true flexibility in the binding mode. The AQP5–ezrin interaction was further evidenced by PLA in hMSG acini. The numerous AQP5–ezrin complexes observed in SICCA-NS hMSG acini were quantitatively lost in SICCA-SS hMSG acini. In addition, qualitative analysis by double immunofluorescence showed that ezrin was mainly localized at the acinar apical membrane in SICCA-NS tissues and co-localized with AQP5 staining. In contrast, the staining of ezrin in SICCA-SS was often missing or weakly mislocalized to the acinar lateral or basal membrane, and AQP5 staining was often mislocated at the basolateral instead of apical pole of acinar cells. Our data on ezrin mislocalization are in agreement with those of another study showing that altered ezrin expression and localization in SS hMSG acini induced a disruption of microvilli architecture and polarity [39]. Therefore, the identification of AQP5–ezrin interaction supports the hypothesis that the altered expression and localization of ezrin may induce altered AQP5 trafficking and lead to deviated end-point AQP5 localization (mostly to the basal, rather than apical, pole of SG acinar cells). Further studies will be required to test this hypothesis and to assess the possible correlation between the number of ezrin reactivity degrees or number of AQP5–ezrin complexes (PLA red dots) and saliva secretion. Considering the complexity of the exocytosis machinery and the possible involvement of other partner proteins such as PIP, the altered expression of AQP5 protein partners may account for AQP5 mislocalization and explain the decreased saliva flow observed in SS patients.

In summary, our data show for the first time a protein–protein interaction between AQP5 and ezrin. Our computer modeling reveals a novel protein domain interaction and a novel peptide consensus involved. Furthermore, our data show a localization of the AQP5–ezrin complexes mostly at the apical side of SICCA-NS hMSG acini and a disruption of the AQP5–ezrin interactions and mislocalization of the protein partners in SICCA-SS hMSG acini. In SICCA-SS hMSG acini, ezrin was mislocalized at the basal or lateral region of acini and showed a significant reduction in the number of AQP5–ezrin complexes as well. Considering the fundamental role of ezrin as a linker between the cytoplasmic membrane and cytoskeleton, its loss mainly in the acinar apical region could be responsible for altered the AQP5 trafficking and mislocalization observed in SS patients. This hypothesis opens new avenues for further studies.

## 4. Materials and Methods

### 4.1. Cell Culture and Transfection

Normal salivary gland-SV40 transformed-squamous cells resembling acinar cells (NS-SV-AC cells; kindly provided by Prof. M. Azuma) [43] were grown and passaged twice a week, as previously described [12]. NS-SV-AC cells that do not express AQP5 endogenously were transfected by electroporation (270 V, 700 μF) using a Gene Pulser II System (Bio-Rad, Hercules, CA, USA) with 8 µg of plasmid. 

### 4.2. Plasmid Preparation

HA-AQP5 and HA-CT plasmids were prepared as previously described [12]. Briefly, an AQP5 cDNA was amplified by PCR from human lung cDNA and then inserted into pcDNA3.1 containing an HA tag (human influenza hemagglutinin) to generate the HA-AQP5 plasmid. The empty vector containing an HA tag in pcDNA3.1 (HA-CT plasmid) was used as a negative control. 

### 4.3. Western Blot Analysis

Total proteins were separated by electrophoresis using 12% SDS-polyacrylamide Tris-Gly Novex precast gels (Thermo-Fisher Scientific, Waltham, MA, USA) and then electrotransferred to a polyvinylidene difluoride (PVDF) membrane. PVDF membrane was incubated with 5% nonfat milk in PBS-0.1% Tween 20 (PBST) for 1 h at room temperature, and then with the primary antibody anti-AQP5 (1:1000; Proteintech, Rosemont, IL, USA) and anti-Actin (1:1000; Millipore, Burlington, MA, USA) in PBST overnight at 4 °C, and finally washed in PBST for 15 min. The PVDF membrane was then incubated with anti-mouse or anti-rabbit antibody (1:3000; Cell Signaling, Danvers, MA, USA) for 1 h at room temperature and washed in PBST. PVDF membranes were incubated with Western Lighting Plus-ECL reagents (Perkin-Elmer, Waltham, MA, USA) and developed using Amersham Imager 600 (GE Healthcare, Chicago, IL, USA).

### 4.4. Stable Isotope Labeling with Amino Acids in Cell Culture (SILAC)-Immunoprecipitation

NS-SV-AC cells were grown in DMEM:F12 medium lacking arginine and lysine supplemented with 10% dialyzed FBS, 2 mM of glutamine, 100 U/mL of penicillin, 100 µg/mL of streptomycin, 1.73 mM of proline, 0.47 mM of light (L medium) or heavy (^13^C6, ^15^N4 Arg; H medium) arginine, and 0.46 mM of light (L medium) or heavy (^13^C6, ^15^N2; H medium) lysine (Thermo-Fisher Scientific, Waltham, MA, USA), and complete labelling was verified by LC MS/MS. After 10 divisions, cells were transfected with HA-AQP5 (HA-AQP5 NS-SV-AC) or HA-CT (HA-CT NS-SV-AC). HA-AQP5 NS-SV-AC and HA-CT NS-SV-AC cells grown each in L and H media were harvested in homogenization buffer (180 mM of Tris containing 0.1 µM of CaCl_2_, 0.8 mM of MgCl_2_, 0.01% SDS, 0.05% sodium deoxycholate, 0.1% Triton X-100, 0.5 mM of NaF, 0.01 mM of vanadate, and cOmplete™ EDTA-free protease inhibitor cocktail (one tablet per 10 mL; Sigma-Aldrich, St-Louis, MI, USA), pH 7.2). Homogenates were mixed for 30 min at 4 °C using a rotating shaker and centrifuged at 17,000× *g* for 20 min at 4 °C. Supernatants were collected prior to protein assay using a Pierce BCA protein assay (Thermo-Fisher Scientific, Waltham, MA, USA). In one experiment, total proteins from HA-AQP5-transfected cells grown in heavy (H) media and from HA-CT-transfected cells grown in light (L) media were mixed at a 1:1 ratio (samples 1 and 3). The labels were swapped in a second experiment to induce inverted H/L ratios as an indicator of specificity (samples 2 and 4). The samples were immunoprecipitated overnight at 4 °C in the absence (negative control) or presence of rabbit anti-AQP5 (Millipore, Burlington, MA, USA) (samples 1 and 2) or mouse anti-HA antibody (Proteintech, Rosemont, IL, USA) (samples 3 and 4) (1 µL per 800 µg of protein), followed by incubation with protein A-coated Sepharose beads (for rabbit antibodies) or protein G-coated Agarose beads (for mouse antibodies) (Sigma-Aldrich, St-Louis, MI, USA) at 4 °C for 1 h. Beads were washed 3 times with homogenization buffer and bound proteins were eluted with 20 µL of Laemmli buffer containing 10 mg/mL of dithiothreitol following 30 min of heating at 37 °C and subsequent centrifugation at 17,000× *g* for 5 min at room temperature. 

### 4.5. Trypsin Digestion of Immunoprecipitated Proteins

NS-SV-AC immunoprecipitated proteins were combined with SDS-PAGE LDS sample buffer containing 50 mM of DTT and loaded onto a Novex Bis-tris gel. Proteins were run into the gel for 15 min, and the gel was stained with Novex colloidal Coomassie stain (Thermo-Fisher Scientific, Waltham, MA, USA) and then destained in water. Gel regions were cut and diced into 1 mm^3^ pieces. Proteins were treated for 30 min with 45 mM of DTT, and available Cys residues were carbamidomethylated with 100 mM of iodoacetamide. Gel pieces were destained with 50% MeCN in 25 mM of ammonium bicarbonate, and proteins were digested with trypsin (10 ng/µL) in 25 mM of ammonium bicarbonate overnight at 37 °C. Peptides were extracted by gel dehydration with 60% MeCN and 0.1% TFA. The extracts were dried by speed vac centrifugation and reconstituted in 0.1% formic acid. Peptides were then analyzed by LC–coupled tandem mass spectrometry (LC-MS/MS). An analytical column was packed with 20 cm of C18 reverse phase material (Jupiter, 3 μm beads, 300 Å, Phenomenex) directly into a laser-pulled emitter tip. Peptides were loaded on the capillary reverse phase analytical column (360 μm O.D. × 100 μm I.D.) using a Dionex Ultimate 3000nanoLC and autosampler. The mobile phase solvents consisted of 0.1% formic acid and 99.9% water (solvent A), and 0.1% formic acid and 99.9% acetonitrile (solvent B). Peptides were gradient-eluted at a flow rate of 350 nL/min, using a 90 min gradient. The gradient consisted of the following: 1–70 min, 2–40% B; 70–78 min, 40–90% B; 78–80 min, 90% B; 80–81 min, 90–2% B; 81–90 min (column re-equilibration), 2% B. A Q Exactive Plus mass spectrometer (Thermo-Fisher Scientific, Waltham, MA, USA), equipped with a nanoelectrospray ionization source, was used to mass-analyze the eluting peptides using a data-dependent method. The instrument method consisted of MS1 scans using an MS AGC target value of 3 × 10^6^, followed by up to 20 MS/MS scans of the most abundant ions detected in the preceding MS scan. The MS2 AGC target was set to 5 × 10^4^, dynamic exclusion was set to 10 s, the HCD collision energy was set to 28NCE, and peptide match and isotope exclusion were enabled. For peptide and protein identification, data were analyzed using the Maxquant software package, version 1.3.0.5. MS/MS spectra were searched against a human subset of the UniprotKB protein database. A multiplicity of 2 was selected for Arg10 and Lys8 SILAC labels, enzyme specificity was set to trypsin, and a maximum of 2 missed cleavages were allowed. Variable modifications included the oxidation of methionine and carbamidomethylation of cysteine. The target-decoy false discovery rate (FDR) for peptide and protein identification was set to 1% for both peptides and proteins. For SILAC protein ratios, a minimum of 2 unique peptides and a minimum H/L ratio count of 2 were required. To obtain ezrin peptide peak intensities, the raw files were imported into Skyline [44] for peak-picking, and quantitation was based on MS1 intensities. The precursor isotopic import filter was set to a count of three (M, M + 1, and M + 2) at a resolution of 60,000.

### 4.6. Proximity Ligation Assay

Proximity ligation assays (PLAs) were performed using Duolink kit (Sigma-Aldrich, St-Louis, MI, USA) according to the manufacturer’s instructions. PLAs were performed on paraffin-embedded hMSG sections using rabbit anti-AQP5 (1:100; Millipore, Burlington, MA, USA) and mouse anti-ezrin (1:100; Thermo-Fisher Scientific, Waltham, MA, USA). PLAs were performed on methanol-fixed transfected NS-SV-AC cells using mouse anti-HA-tag (1:100; Proteintech, Rosemont, IL, USA) and rabbit anti-ezrin (1:200; Cell Signaling, Danvers, MA, USA). Negative controls were performed in the absence of one or both antibodies. Z-stack images were acquired using a confocal microscope (LSM-710) with an ×63/1.4 PlanApochromat lens (Zeiss, Oberkochen, Germany) and processed as previously described [12].

### 4.7. Docking Simulations

As the distal part of the C-terminus is disordered in the crystal structure of human AQP5, the structure of the full AQP5 C-terminus was predicted using Robetta [34]. Two approaches were used, one using comparative modeling based on the existing human AQP5 structure [35] (PDB code 3D9S), and the other with de novo structure prediction in TrRosetta using machine learning. Both models were then paired with the structure of the active FERM-domain of ezrin (PDB:1NI2) and put through initial docking trials using PyRosetta [36]. Here, 1000 decoys were generated, scored, and analyzed. Well-scoring decoys were inspected and further analyzed using HADDOCK 2.4 [37]. The highest scoring clusters were manually inspected using PyMOL (PyMOL Molecular Graphics System, Schrödinger, LLC), and further analyzed using PRODIGY [38,45] along with other structurally characterized complexes between FERM-domains and helical peptides: *Mus musculus* Radixin FERM-domain in complex with NHERF-1 (PDB code 2D10) and NHERF-2 (PDB code 2D11) C-terminal tail peptides [46], *Drosophila melanogaster* Merlin FERM-domain in complex with the Merlin C-terminus (PDB code 7EDR) [46], and the human Moesin FERM-domain in complex with EBP50 (also known as NHERF-1) C-terminal peptide (PDB code 1SGH) [47]. Residues involved in the interaction were initially compared through visual alignment, and finally via multiple sequence alignment using Clustal Omega [48] in order to elucidate the level of conservation across the various proteins.

### 4.8. Human Minor Salivary Gland Samples

Paraffin-embedded human minor salivary gland biopsies (hMSG) archived in the Erasme Hospital Biobank (Brussels, Belgium; BE_BERA1; Biobanque Hôpital Erasme--ULB (BERA); BE_NBWB1; Biothèque Wallonie Bruxelles (BWB); BBMRI--ERIC) were sectioned at a 6 µm thickness by Diapath (part of the Center for Microscopy and Molecular Imaging (CMMI)). Biopsies were performed at the time of diagnosis. Patients with sicca symptoms (without signs of autoimmunity) and primary SS (SICCA-SS; *n* = 23; 61 ± 3 years old) fulfilled the American College of Rheumatology (ACR)/European League against Rheumatism (EULAR) classification criteria for the disease [49] and had a focus score ≤1. Patients with nonspecific sialoadenitis but no SS (SICCA-NS) were used as negative controls (*n* = 19; 69 ± 2 years old). The study was approved by the ULB Erasme Hospital ethics committee (P2016/299).

### 4.9. Double Immunofluorescence

Double immunofluorescence was performed on deparaffined and permeabilized hMSG sections using rabbit anti-AQP5 (1:100; Millipore, Burlington, MA, USA), mouse anti-ezrin (1:100; Thermo-Fisher Scientific, Waltham, MA, USA), anti-rabbit antibody-conjugated Alexa 488 (1:1000; Cell Signaling, Danvers, MA, USA), and biotinylated anti-mouse (1:200; Jackson ImmunoResearch, West Grove, PA, USA) followed by a streptavidin-anti-mouse conjugated-Alexa594 (1:100; Sigma-Aldrich, St-Louis, MI, USA). Immunofluorescent labeling of ezrin was quantified on the images captured at 20× magnification using a Leica DM 2000 microscope. One microscopic field, generally containing the whole section, was analyzed for each sample. Tissue containing acini was selected and the reacting surfaces were quantified using CellSens Imaging Software (Olympus, Düsseldorf, Germany). The color threshold was calculated on negative controls. Image analysis was performed using the percentage of the reacting area and the level of pixel color intensity per field. The degree of ezrin reactivity was calculated as the product between the average of the positive area percentage and the mean value of pixel color intensity per microscopic field.

### 4.10. Statistical Analysis

The Shapiro–Wilk test (test of normality), Student’s *t*-test, and Mann–Whitney U test were performed using IBM SPSS Statistics 25. Data are expressed as mean ± S.E.M. of n experiments. Data are considered significant when *p* < 0.05.

## Figures and Tables

**Table 1:** HADDOCK docking statistics for the two best clusters for the AQP5 C-terminus modeled by Comparative Modeling (*CM*) *TrRosetta*. All energies are given in kcal/mol. Root-mean-square deviation (RMSD) is calculated in relation to the overall lowest-energy structure of the cluster. The Z-score indicates how many standard deviations from the average the cluster is in terms of score (the more negative, the better).

	CM Cluster 1	±	CM Cluster 4	±	CM Cluster 2	±	CM Cluster 1	±
HADDOCK score	−94.5	5.8	−91.9	8.3	−91.2	5.1	−89.8	4.7
Cluster size	46		13		22		25	
RMSD (Å)	16.0	0.5	21.4	0.6	18.3	0.5	17.9	0.3
Van der Waals energy	−16.9	5.3	−7.8	5.9	−13.1	2.2	−9.4	6.0
Electrostatic energy	−488.1	26.9	−537.4	34.6	−373.6	34.7	−531.0	20.9
Desolvation energy	17.8	1.8	22.4	1.7	−4.9	1.9	19.8	2.1
Restraints violation energy	21.6	19.4	11.3	17.7	15.3	17.6	59.8	36.9
Buried surfaces (Å2)	1287.3	39.4	1178.9	55.2	1181.3	36.7	1201.4	56.9
Z-score	−1.5		−1.4		−1.0		−0.9	

**Table 2:** PRODIGY interaction statistics for Cluster1_1-1_4 (model 1, comparative modeling), Cluster2_1-2_4 (model 2, fTrRosetta), and crystal structures of other complexes between alpha helical peptides and FERM domains (2D11 and 2D10: Mus musculus radixin FERM with NHERF-2 and NHERF-1 peptides, respectively; 7EDR: Drosophila melanogaster Merlin FERM with Merlin C-terminal domain; 4ZRJ: Human Merlin with C-terminal domain; 1SGH: Human Moesin FERM with EBP-50 (also known as NHERF-1).

	C1_1	C1_2	C1_3	C1_4	C2_1	C2_2	C2_3	C2_4	2D11	2D10	7EDR	4ZRJ	1SGH
ΔG (kcal mol^−1^)	−6.6	6.9	−6.4	6.2	−6.7	−6.5	−6.4	−6.1	−8.2	−8.2	−9.8	−6.5	−4.8
ICs charged-charged:	15	15	15	15	12	10	11	11	5	2	4	5	0
ICs charged-polar:	9	9	6	6	3	2	4	3	4	5	11	5	3
ICs charged-apolar:	10	11	11	8	21	20	19	16	25	21	10	13	2
ICs polar-polar:	0	0	0	1	0	0	0	0	2	2	0	0	2
ICs polar-apolar:	3	4	2	3	0	0	0	0	6	8	12	3	4
ICs apolar-apolar:	3	3	3	4	4	4	6	3	27	26	13	22	5
NIS charged:	26.21%	26.26%	26.02%	26.44%	25.33%	25.67%	25.45%	25.50%	38.11%	37.70%	32.35%	38.68%	34.50%
NIS apolar:	47.36%	47.26%	47.96%	46.90%	48%	47.54%	47.99%	47.89%	34.43%	33.61%	30.88%	34.98%	27.98%

## Data Availability

The data presented in this study are available on request from the corresponding authors.
